# Positive Healthy Organizations: Promoting Well-Being, Meaningfulness, and Sustainability in Organizations

**DOI:** 10.3389/fpsyg.2017.01938

**Published:** 2017-11-14

**Authors:** Annamaria Di Fabio

**Affiliations:** Department of Education and Psychology, Psychology Section, University of Florence, Florence, Italy

**Keywords:** healthy organizations, healthy business, positive psychology, occupational health psychology, positive organizational health psychology

## Abstract

This contribution deals with the concept of healthy organizations and starts with a definition of healthy organizations and healthy business. In healthy organizations, culture, climate, and practices create an environment conducive to employee health and safety as well as organizational effectiveness (Lowe, 2010). A healthy organization thus leads to a healthy and successful business (De Smet et al., 2007; Grawitch and Ballard, 2016), underlining the strong link between organizational profitability and workers’ well-being. Starting from a positive perspective focused on success and excellence, the contribution describes how positive organizational health psychology evolved from occupational health psychology to positive occupational health psychology stressing the importance of a primary preventive approach. The focus is not on deficiency and failure but on a positive organizational attitude that proposes interventions at different levels: individual, group, organization, and inter-organization. Healthy organizations need to find the right balance between their particular situation, sector, and culture, highlighting the importance of well-being and sustainability. This contribution discusses also the sustainability of work-life projects and the meaning of work in healthy organizations, stressing the importance of recognizing, respecting, and using the meaning of work as a key for growth and success. Finally, the contribution discusses new research and intervention opportunities for healthy organizations.

## Introduction: Healthy Organizations and Healthy Business

The World Health Organization’s (WHO) primary function is to improve working conditions as occupational health is closely associated with public health (World Health Organization, 2007). The WHO is interested in factors impacting workers’ health such as risks of disease and injury in the occupational environment, social and individual factors, and access to health services. The WHO proposed a Global Plan of Action on Workers’ Health 2008–2017, which was endorsed by the World Health Assembly in 2007 with the following objectives: devising and implementing policy instruments for workers’ health, promoting health in the workplace, improving the performance of and access to occupational health services, providing and communicating information for action and practice, and incorporating workers’ health into other policies.

Work plays a key role in the health and well-being of workers, and it is important to recognize the negative impact on workers of the current world of work characterized by globalization and technology advances (Sparks et al., 2001). As a consequence of globalization, workers today experience greater job insecurity as well as the negative effects of the introduction of information technology such as long hours of work at visual display terminals, which can be detrimental to their health (Sparks et al., 2001). Robots and other computer-assisted technologies taking over tasks previously performed by human beings adds to workers’ concern about the future of jobs and wages (Acemoglu and Restrepo, 2017; Blustein et al., 2017). Both the psychological and physical well-being of workers is thus under threat. The instability and insecurity in today’s world of work calls for the promotion of healthy organizations and healthy business as part of a primary prevention approach (Hage et al., 2007; Kenny and Hage, 2009; Di Fabio and Kenny, 2015, 2016). A major challenge in the 21st century is to create healthier societies by promoting healthy organizations (Di Fabio, 2017; Di Fabio et al., 2017).

In healthy organizations, culture, climate, and good practices create an environment that can promote employee health and safety as well as organizational effectiveness (Lowe, 2010). A healthy organization is conducive to healthy and successful business (De Smet et al., 2007; Grawitch and Ballard, 2016) thus underlining the strong link between organizational profitability and workers’ well-being (Raya and Panneerselvam, 2013; Arnoux-Nicolas et al., 2016). Grawitch and Ballard (2016), too, maintain that a healthy organization is not only an organization that makes good profits but an organization that also promotes a healthy business environment through the well-being of workers.

## The Positive Perspective

From a positive psychology point of view (Seligman and Csikszentmihalyi, 2000; Seligman, 2002; Di Fabio, 2016), the four factors in a healthy organization that need to be considered are the individual, the group, the organization, and inter-organizational processes (Henry, 2005).

At the individual level, interventions to improve the psychological health of the employees and the organization as a whole should be introduced. In particular, it is necessary to enrich jobs, improve employees’ motivation, provide feedback, and increase employee participation (Judge et al., 2001; Henry, 2005; Di Fabio, 2017). Interventions are aimed at building strengths (Di Fabio, 2014a; Di Fabio and Kenny, 2015), enhancing positive individual resources such as emotional intelligence and resilience (Di Fabio and Saklofske, 2014a; Di Fabio, 2015), and promoting well-being (Di Fabio and Saklofske, 2014b; Di Fabio, 2015; Di Fabio and Kenny, 2015). Interventions aimed at bringing about personal development, confidence, and forgiveness enhance psychological maturity and can help employees interact with each other in a healthier and more productive manner (Judge et al., 2001; Henry, 2005; Di Fabio, 2017).

At the group level, a healthy group is a group that respects its members, takes time to listen to their views, tolerates different styles, and aims for win-win solutions. The focus is on team building (belonging to a team is central to most people’s sense of well-being), group training (promotes identifying, accepting, and working with diversity), creative thinking (healthy groups are open to creative challenges from members) (Carter and West, 1999; Henry, 2005; Di Fabio, 2017), and workplace relational civility (Di Fabio and Gori, 2016) in terms of relational decency, relational culture, and relational readiness for positive interactions with other employees, which can reduce conflict in organizations. Interventions aimed at creating healthy groups can help employees build strong bonds and the social support needed to face the complexities of today’s world of work and preserve a sense of well-being (Carter and West, 1999; Henry, 2005; Di Fabio, 2017).

At the organization level, healthy organizations, too, are open to challenges. The focus is on making the organization a more efficient and happy place to work in and more competitive in the global world of work, creating an open culture characterized by sustained creativity and innovation, and promoting an organizational climate that supports positive relationships and leadership styles for the empowerment of employees through autonomy and self-organization (Taylor, 2002; Henry, 2005; Tetrick and Peiró, 2012; Di Fabio, 2017; Di Fabio et al., 2017).

At the inter-organization level, the focus is on making the boundaries of organizations more fluid and improving the relations between organizations. Partnerships, networking, and community involvement are important here (Stacy, 1996; Henry, 2005; Di Fabio, 2017). At this level, it is important to promote partnerships between organizations across the supply chain for their mutual benefit. It is also important to facilitate individual networking of employees within, outside, and across organizations to improve performance and business prospects. Also of importance are community programs that involve employees in some form of community work such as teaching the underprivileged, renovating buildings, etc. (Stacy, 1996; Henry, 2005; Di Fabio, 2017).

## Occupational Health Psychology

The term “occupational health psychology (OHP)” was coined at the University of Hawaii (Raymond et al., 1990) with the focus on healthy workplaces (Quick et al., 1997) where people could produce, serve, grow, be valued, and use their talents and gifts to achieve high performance, high satisfaction, and well-being. Two OHP societies were later established: one in Europe and one in the United States. In 1999, the European Academy of Occupational Health Psychology was founded in Nottingham (United Kingdom) with the aim of applying psychology to occupational health (Cox et al., 2000). In 2004, the Society for Occupational Health Psychology (SHOP) was established in Portland (United States) with the aim of conducting psychological research on the health of workers and their problems in the workplace. Tetrick and Peiró (2012) state that in the mid-1990s OHP introduced a balanced approach to well-being and efficiency with the aim of improving the quality of work-life for workers. OHP’s definition of health is consistent with that of the WHO, where health is seen not simply as the absence of illness (Tetrick and Peiró, 2012) but as optimal functioning (Tetrick, 2002; Hofmann and Tetrick, 2003; Tetrick et al., 2005). Tetrick and Peiró (2012) add that OHP extends the conceptualization of safety to include psychosocial factors in the work environment such as climate, interpersonal relations, co-workers’ support, and leadership. They stress the importance of recognizing the value of and integrating a positive approach into the realities of today’s work environment (Tetrick and Peiró, 2012).

Occupational health psychology promotes a primary prevention approach (Tetrick and Peiró, 2012), focusing traditionally on the elimination of risks to employees’ safety and health (Quick and Tetrick, 2003) and more recently on the promotion of positive experiences, particularly the development of safe and healthy work environments (Kelloway et al., 2008).

## Positive Organizational Health Psychology

A positive primary preventive approach (Di Fabio and Kenny, 2016; Di Fabio et al., 2016) can be fostered in organizational contexts based on efforts to increase employees’ resources (Seligman, 2002; Di Fabio et al., 2014, 2016). Primary prevention (Caplan, 1964) stresses the importance of preventing the development of a problem before it starts and of promoting psychological well-being. The focus is thus on building the strengths of employees/workers.

Positive organizational health psychology (Di Fabio, 2017) developed from a positive primary preventive perspective (Di Fabio and Kenny, 2016; Di Fabio et al., 2016) with the focus on enhancing and promoting resources and talents with interventions at the four levels discussed earlier: the individual, the group, the organization, and the inter-organization level. According to this perspective, healthy organizations need to create the right balance in their particular situation, sector, and culture, highlighting the importance of well-being and sustainability (Di Fabio, 2017). The challenge facing us today is to promote a healthier society by building healthy organizations with the focus on well-being from a cross-cultural perspective (Di Fabio, 2017).

The psychology of sustainability (Di Fabio, 2017) covers the issue of positive sustainable organizational development in a culturally diverse world (Akay et al., 2017; Di Fabio, 2017). Here the attention is on both hedonic well-being (Watson et al., 1988) and eudaimonic well-being (Ryan and Deci, 2001; Waterman et al., 2010). Hedonic well-being comprises an affective evaluation in terms of positive and negative affects (Watson et al., 1988) and a cognitive evaluation in terms of life satisfaction (Diener et al., 1985). Eudaimonic well-being concerns optimal functioning and self-realization (Ryan and Deci, 2001), life meaning and purposefulness (Waterman et al., 2010), and positive functioning (Ryff, 1989). Because meaningfulness is integral to sustainability (Di Fabio and Blustein, 2016), employees need to experience hedonic well-being and especially eudaimonic well-being in order to recognize the deepest meanings and authentic aspects of the Self, which can lead to a real sense of accomplishment and full self-realization as major forms of well-being. Meaningfulness represents the intrinsic motivational energy that promotes real sustainability for employees and their projects, performances, developments, and choices (Di Fabio, 2017).

Disabato et al. (2016) study of Diener’s (1984) subjective well-being model revealed a strong relationship between hedonic well-being and happiness, pleasure, and engagement, while Ryff’s (1989) psychological well-being model posits a strong relationship between hope, life meaning, and determination (goal-directed behavior). Both hedonic and eudaimonic well-being reveal similar relationships with curiosity and gratitude.

Positive organizational health psychology calls for an organizational approach centered on enhancing resources and building strengths and not on deficiency and failure from a primary prevention point of view (Hage et al., 2007; Kenny and Hage, 2009; Di Fabio and Kenny, 2015, 2016). It thus calls for early interventions aimed at increasing both the hedonic and eudaimonic well-being of workers at different levels (individual, group, organization, and inter-organization) to promote healthy organizations.

## Sustainability of Work-Life Projects and Meaning for Healthy Organizations

The concept of the sustainability of work-life projects in terms of coherence, direction, significance, and belonging was developed as part of promoting well-being and healthy organizations (Schnell et al., 2013; Di Fabio, 2017). Here it is important to stress the shift from a motivational paradigm to a meaning paradigm (Di Fabio and Blustein, 2016; Di Fabio, 2017). A motivational paradigm concerns motivation and highlights intrinsic motivation in terms of doing a job to gain satisfaction; extrinsic motivation in terms of doing a job for reward or to avoid punishment; and lack of motivation in terms of lack of perception of the link between behavior and its consequences in the workplace (Tremblay et al., 2009; Deci and Ryan, 2010). The meaning paradigm (Di Fabio and Blustein, 2016) goes further: it posits the centrality of meaning in understanding how people can establish meaningful lives and meaningful work experiences, and links the sustainability of life-work projects to meaningful construction in their lives. The meaning paradigm is thus key to the sustainability, growth, success, and health of organizations (Di Fabio, 2017). Positive organizational narratives are essential for ensuring sustainable development in organizations (Di Fabio, 2017). Such narratives often appear complex and confusing, but they can be transformed into coherent stories that produce meaning, hope, possibilities, and success for healthy organizations (Di Fabio, 2017). These narratives can also be linked to the culture of each employee thus introducing a new positive perspective in a diversity management framework (Cox, 2001) where the organizational culture is transformed from a culture oriented to the majority to a culture that accommodates different value systems that impact on the work environment. This promotes the recognition of diversity as an opportunity to increase performance and new points of view for a healthy business.

The importance of a quali + quanti approach (Di Fabio and Maree, 2013) is that details of meaning are used to help construct and implement optimal stories starting with concrete real-life work situations and ending with a focus on relationships, meaning, and details of meaning (Blustein, 2011, 2013; Di Fabio and Blustein, 2016; Di Fabio, 2017).

The “storied self” (Savickas, 2005, 2011) has developed from three different perspectives (Di Fabio, 2017). The first perspective “from facts” is based on grounded reflexivity (Guichard, 2004, 2005; Di Fabio, 2014b; Di Fabio and Maree, 2016), which is a process contained in the formula: “reflexivity in, on, for” as reflexivity is a dynamic and continuous process of self-awareness (Finlay and Gough, 2003; Guichard, 2004, 2005; Maree, 2013). The three levels of reflexivity are (Maree, 2013): reflection-in-action, that is, reflection on certain issues during the action or while the person acts; reflection-on-action, that is, retrospective thought, thought after an action or an event; reflection-for-action, that is, reflection before a particular action. Reflectivity refers thus to the capacity to analyze the present and to look at the past, individuating significant life themes of use in constructing a bridge toward the future (Di Fabio and Maree, 2016).

The second perspective “from perception of the facts” involves considering narrative identity (Guichard, 2004, 2010; Savickas, 2011, 2015), which is based on the concepts of Self as story, narratability, and biographicity (Guichard, 2010; Savickas, 2011). Through the stories of their different life experiences and their future plans, people can give meaning to their lives, develop their own identities and their own Self, and give meaning to their existence (Savickas, 2011). Narrative identity is thus “a person’s internalized and evolving life story, integrating the reconstructed past and imagined future to provide life with some degree of unity and purpose” (McAdams and McLean, 2013, p. 233). Better adapted people tend to tell stories in which they find redemptive meaning in suffering and adversity and construct life stories that feature themes of personal agency and exploration (McAdams and McLean, 2013). They tend also to achieve higher levels of mental health, well-being, and maturity (McAdams and McLean, 2013). In the narrative process, it is therefore important to facilitate the emergence of positive narratives, transforming negative stories about employees and about organizations into positive stories that enable employees to construct new ways to build their own new positive future reality.

The third perspective “from success experience” covers narrative success (Guichard, 2010; Savickas, 2011; Di Fabio, 2016) and narrative details of meaning (Di Fabio, 2017) with the emphasis on experiences of success and the achievement of success through relationships involving the worker, the team, and the organization (Di Fabio, 2017). By relating stories of success, employees can focus on positive experiences regarding their performance resulting in positive energizing psychological effects in terms of self-esteem and self-efficacy. They can then also more easily face new challenges by recognizing personal positive resources to construct new chapters of their successful lives thereby enhancing their well-being.

It is important to act timeously to strengthen the worker, the team, and the organization by focusing on positive work experiences in today’s changeable and competitive market place (Di Fabio, 2017). Organizational practices aimed at achieving positive work experiences and positive psychological narratives at work are a key part of a primary prevention approach (Di Fabio, 2017).

## Conclusion

Positive healthy organizations are based on building resources and strengths with success as the criterion. A positive approach is adopted toward individuals, groups, and organizations as part of an early primary prevention intervention. The innovation of focusing on experiences of success in relationships between workers, teams, and organizations (Di Fabio, 2017) could open new opportunities for research and intervention. In fact, such relationships could be a central feature of healthy organizations (Blustein, 2006, 2011) and of new ways of conceptualizing organizational relationality. This refers not only to prosocial organizational behavior, organizational citizenship behavior, organizational support, organizational welfare, but also to the new construct of workplace relational civility (Di Fabio and Gori, 2016) that includes relational decency, relational culture, and relational readiness. Also, some current innovative leadership styles can make a significant contribution to healthy organizations (Clark, 2012; Hoffmeister et al., 2014). Ethical (Gallagher and Tschudin, 2010), sustainable (Hargreaves and Fink, 2004), and servant leadership (Ehrhart, 2004) can promote the development of healthy organizations. Ethical leadership aspires to strive for ethical goals and to empower members of the organization, emphasizing employees’ strengths rather than their weaknesses (Gallagher and Tschudin, 2010). Sustainable leadership refers to the shared responsibility not to exhaust the organization’s human and financial resources and to restrict social and environmental damage as far as possible (Hargreaves and Fink, 2004). And servant leadership refers to the premium placed on the personal growth and well-being of subordinates in the organization (Greenleaf, 2002). These leadership styles focus on promoting the resources, talents, and potential of employees thereby enabling them to realize themselves fully and achieve well-being as part of healthy organizations.

Recently, the concept of health-promoting leadership (Jiménez et al., 2016) was developed as a leadership style for creating conditions that enhance employee health in a healthy work environment. A new awareness is needed in organizational contexts of the value of developing early interventions and new approaches from a primary preventive perspective to foster healthy work environments. Enhancing the resources, strengths, and talents of workers and groups is the best way to achieve well-being and healthy workplaces. This calls for acknowledging the importance of relationships and meaning (Blustein, 2006, 2011, 2013; Di Fabio and Blustein, 2016) in constructing positive organizational narratives and thereby promoting healthy organizations.

## Author Contributions

ADF ideated the structure, analyzed the literature, and wrote the manuscript.

## Conflict of Interest Statement

The author declares that the research was conducted in the absence of any commercial or financial relationships that could be construed as a potential conflict of interest.
